# Regulation and mechanism of YAP/TAZ in the mechanical microenvironment of stem cells

**DOI:** 10.3892/mmr.2021.12265

**Published:** 2021-06-30

**Authors:** Ying Li, Jinming Wang, Weiliang Zhong

Mol Med Rep 24: Article no. 506, 2021; DOI: 10.3892/mmr.2021.12145

Owing to an error that was made during the production stages of the above review article, what was actually Fig. 2 was inadvertently duplicated on p. 7 as Fig. 9. Fig. 9 as it should have appeared in the review is shown below. The Editor apologizes to the authors for this error, and regrets any inconvenience caused to the readership.

## Figures and Tables

**Figure 9.**
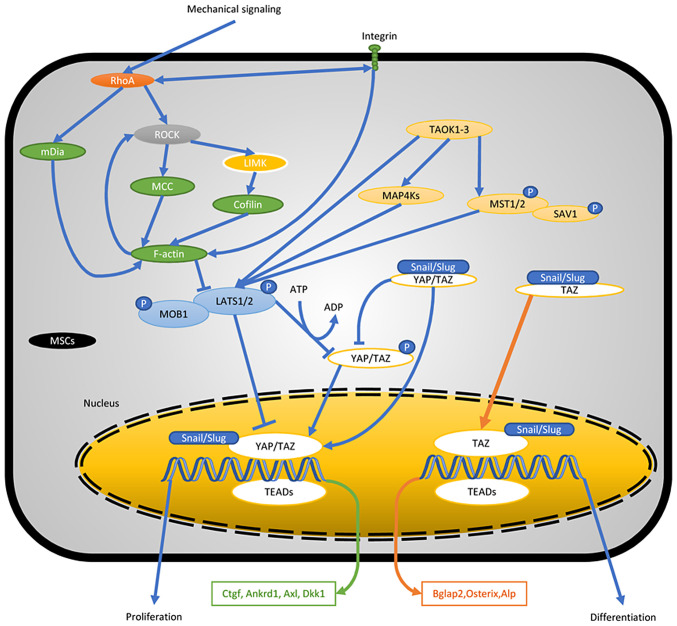
YAP and TAZ mechanotransduction in stem cell biology. A variety of mechanisms of YAP/TAZ regulation via the Hippo pathway and cross-talk with other signaling pathways have been identified. YAP/TAZ, yes-associated protein/transcriptional coactivator with PDZ-binding motif; F-actin, filamentous actin; TAOK, TAO kinase; SAV, Salvador family; WW-domain-containing protein; LATS, large tumor suppressor kinase; MOB, MOB kinase activator; TEAD, the transcriptional enhanced associate domain; MAP4K4, mitogen-activated protein kinase kinase kinase kinase 4; MST1/2, macrophage stimulating 1/2.

